# High-resolution data on the impact of warming on soil CO_2_ efflux from an Asian monsoon forest

**DOI:** 10.1038/sdata.2017.26

**Published:** 2017-03-14

**Authors:** Naishen Liang, Munemasa Teramoto, Masahiro Takagi, Jiye Zeng

**Affiliations:** 1 Center for Global Environmental Research, National Institute for Environmental Studies, Tsukuba, Ibaraki 305-8506, Japan; 2 Faculty of Agriculture, University of Miyazaki, 11300 Tano-cho, Miyazaki 889-1702, Japan

**Keywords:** Climate-change impacts, Ecophysiology, Forest ecology

## Abstract

This paper describes a project for evaluation of global warming’s impacts on soil carbon dynamics in Japanese forest ecosystems. We started a soil warming experiment in late 2008 in a 55-year-old evergreen broad-leaved forest at the boundary between the subtropical and warm-temperate biomes in southern Japan. We used infrared carbon-filament heat lamps to increase soil temperature by about 2.5 °C at a depth of 5 cm and continuously recorded CO_2_ emission from the soil surface using a multichannel automated chamber system. Here, we present details of the experimental processes and datasets for the CO_2_ emission rate, soil temperature, and soil moisture from control, trenched, and warmed trenched plots. The long term of the study and its high resolution make the datasets meaningful for use in or development of coupled climate-ecosystem models to tune their dynamic behaviour as well as to provide mean parameters for decomposition of soil organic carbon to support future predictions of soil carbon sequestration.

## Background & Summary

Global soil organic carbon (SOC) pools in the top 100 cm of the soil total an estimated 1,460 to 1,550 Gt (refs 1,2), almost double the amount of carbon in the atmosphere and three times that in global biomass carbon pools^3^. Soil microbiota decompose SOC and emit CO_2_ as heterotrophic respiration (*R*_h_). Soil CO_2_ efflux (*R*_s_) is composed of *R*_h_ and root respiration (*R*_r_), and *R*_s_ is the largest CO_2_ emission in terrestrial ecosystems. Global *R*_s_ totalled 98±12 Gt C in 2008 (ref. 4), and *R*_h_ contributed about 70% of *R*_s_ (ref. 5).

Soil temperature and soil moisture are the primary abiotic factors that control the pattern and magnitude of *R*_s_ (ref. 6). The sensitivity of *R*_s_, *R*_h_, and *R*_r_ to temperature is generally represented using *Q*_10_, an exponential function that expresses the change per 10 °C temperature increase^7^. Recently, Todd-Brown *et al.*^8^ used twelve CMIP5 Earth System Models to reveal high global variation of *Q*_10_ values for *R*_h_ (ranging from 1.45 to 2.61). The high temperature sensitivity of *R*_h_ suggests that even a slight temperature increase caused by climate change will dramatically increase global SOC decomposition, potentially converting the terrestrial carbon sink into a net carbon source after the mid-21st century^9^. However, conflicting results from field experiments showed that the stimulatory effect of warming on *R*_s_ disappeared after 1 or more years^10,11^. That unexpected result was explained by physiological thermal adaptation^12^, depletion of labile SOC^13^, changes in the microbiota species composition^14^, and soil moisture decreases caused by the warming^15^.

Asian monsoon regions, influenced by the Tibeto-Himalayan Plateau, have sufficient rainfall and lack a subtropical dry belt. Thus, Asian monsoon forests have higher net primary production than other ecosystems at the same latitudes and consequently accumulate abundant SOC^16–19^. Thus, the response of *R*_h_ in Asian monsoon forests to global warming is expected to have important feedback on regional and global climate change^20–22^. For example, about 68% of Japan’s land area is covered by forests (25×10^6^ ha; 40% are plantations and 60% are natural forests), and Japan’s plantations sequester about 10.4 Mt C yr^−1^; this is about 3.8% of its national emission reduction commitment (6%) for the ‘First Commitment Period’ (2008 to 2012) of the Kyoto Protocol^23^. However, as these plantations mature and become over-mature, their carbon sequestration may decrease by about 27% (7.6 Mt C yr^−1^) by 2030 (ref. 23).

As a part of a project to evaluate the potential carbon sink or source strength of Japan’s forest soils and its response to climate change, we conducted a soil warming experiment in a 55-year-old evergreen broad-leaved forest at the boundary between southern Japan’s subtropical and warm-temperate biomes starting in December 2008. We increased the soil temperature at a depth of 5 cm by about 2.5 °C using infrared carbon-filament heat lamps, and continuously monitored CO_2_ emission from the soil surface and related environmental parameters with a multichannel automated chamber system. In our previous work^22^, we presented the seasonal and annual trends of *R*_s_, *R*_h_, and *R*_h_ in a warmed trenched treatment (*R*_hw_). Between 2009 and 2014, annual *R*_s_, *R*_h_, and *R*_hw_ averaged 15.7 (with a range from 13.4 to 18.4), 11.9 (10.3 to 13.3), and 13.8 (12.0 to 15.1) tC ha^−1^, respectively. The annual *R*_hw_ was 7.1 to 17.8% (per degree) greater than in *R*_h_ (an average of 9.4% °C^−1^). In addition, the mean annual *Q*_10_ values ranged from 2.34 to 2.97 for *R*_s_, 2.36 to 2.90 for *R*_h_, and 2.23 to 3.02 for *R*_hw_. The annual soil efflux, stimulatory warming effect, and *Q*_10_ values were all positively related to the total summer precipitation^22^.

The objective of this paper is to present a full description of the experimental processes and the high-resolution datasets. Specifically, we provide details about the data collection and suggestions for reuse of the time-series datasets. These datasets will be useful for analysing the changes in soil CO_2_ efflux components on different time scales (from hourly to annual) and the responses of each respiration process to dynamic changes of environmental factors such as soil temperature and moisture.

## Methods

### Site description

We conducted this study at the Miyazaki Experimental Warming Site of the Miyazaki University Forest, in a 55-year-old evergreen broad-leaved forest at the boundary between subtropical and warm-temperate biomes (31°51' N, 131°18' E; 130 m asl) in Kyushu, southern Japan. The dominant species are *Castanopsis cuspidata* and *Machilus thunbergii*. The forest understory is lack of herbaceous vegetation but dominated by a small shrub species of *Eurya japonica*. In 2014, the stand density, mean tree diameter at breast height (DBH), and canopy height were 1,175 stems ha^−1^, 16.5 cm, and 18.0 m, respectively. The basal area totalled 38.3 m^2^ ha^−1^. Between 2009 and 2014, mean annual temperature and precipitation were 17.6 °C and 2,604 mm, respectively. Mean monthly temperatures ranged from 7.0 °C in January to 27.9 °C in August.

The soil is a moderately moist brown forest soil developed from volcaniclastic sediment. The SOC density in the top 30 cm of the soil averaged 9.92 kg m^−2^. In 2014, after the 6-year warming treatment, the SOC density in the top 5 cm of the soil in control and warming treatment were estimated to be 2.23 and 2.09 kg m^−2^, respectively^22^.

### Experimental design

This project focused on the responses and feedbacks of forest SOC to global warming. Root respiration is generally driven by the aboveground vegetation phenology and by photosynthetic activity^24,25^. However, it is nearly impossible to adopt an appropriate approach to raise the temperature of the mature forest canopy^26^. Thus, our experiment was designed to focus on soil factors, and used three treatments: control plots to measure *R*_s_, root trench plots to measure *R*_h_, and trenched plots combined with warming to measure the warming-manipulated *R*_h_ (i.e., *R*_hw_). Here, we are presenting expanded descriptions of the experimental processes and data that were described in our previous work^20–22^.

### Warming

Open-top chambers, resistance heating cables (RHCs), and infrared heat lamps are the three major warming approaches that are used to increase air and soil temperatures in experimental warming manipulations in the field. Each of these approaches has certain advantages and caveats. Open-top chambers are a passive warming tool, and have been widely used for vegetation with a short canopy, such as polar and alpine ecosystems, but the approach leads to non-homogeneous temperatures and soil moisture contents inside the chamber^27^. Buried RHCs are commonly used for ecosystem warming experiments because of their relatively low electricity consumption and lack of fire risk^11^. However, their installation strongly disturbs the soil and the soil biota (an effect that generally lasts for 6 to 12 months), and creates a temperature profile that differs from that in the natural soil; as a result, the use of RHCs is becoming less common^28^.

**Figure 1:**
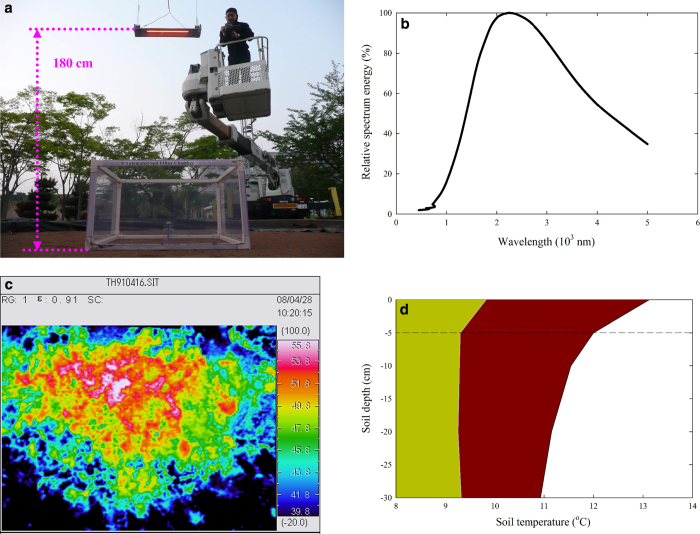
Test of the infrared carbon-filament heat lamp. (**a**) Photograph of the field test. (**b**) Spectrum of the radiation produced by the heat lamp. (**c**) Thermal image of the soil inside the enclosure created using infrared thermography. (**d**) Soil temperature profiles in the control (green) and warming (red) plot. The authors affirm that the individual depicted herein provided informed consent for the use of their image.

Our project used infrared carbon-filament heat lamps to warm the forest soil. This protocol has a high electricity consumption cost, but minimizes the ecosystem disturbance caused by the heating manipulation^20–22^. Compared with buried RHCs, the infrared lamps create an effect similar to the natural global warming process: radiation from the lamps initially heats the air and the soil surface, then the heat energy transfers into the deep soil. Before our warming experiment began, we conducted a field test in April 2008 at the campus of Japan’s National Institute for Environmental Studies (Fig. 1). The target soil warming level of +2.5 °C at a depth of 5 cm was chosen based on the range of warming predicted to occur by approximately 2100 (ref. 3). An 800-W carbon-filament heat lamp (Sakaguchi E.H. VOC. Corp., Akihabara, Tokyo) was suspended at 1.8 m above the soil (Fig. 1a). The lamp was a resistive carbon filament (40 cm long, 5 mm wide, and 0.5 mm thick) enclosed in a glass vacuum tube (1 cm in diameter and 40 cm in length). Its peak wavelength was 2,200 nm (Fig. 1b). An arched stainless reflector 12 cm wide and 40 cm long spreads the radiation evenly over a surface area of about 1.5×2.0 m, and creates a similar temperature profile throughout the top layers of the soil. The temperature distribution at the soil surface was confirmed by means of infrared thermography (Fig. 1c; TH9100ML, Nippon Avionics Co., Ltd., Shinagawa, Tokyo, Japan). Soil temperatures at the surface and at depths of 5, 10, 20, and 30 cm below the surface in the warming plot were 3.0, 2.5, 2.0, 1.7 and 1.5 °C higher, respectively, than in the control soil (Fig. 1d). To maintain a constant soil temperature profile difference between the control and warming plots, the lamps were kept on during the whole experimental period. In our previous study in a subtropical forest in southwestern China, we extensively analysed the soil temperature profiles, and found that a temperature increase of 0.5 °C was still detectable at a depth greater than 1 m in the soil^29^.

### Fire risk

**Figure 2:**
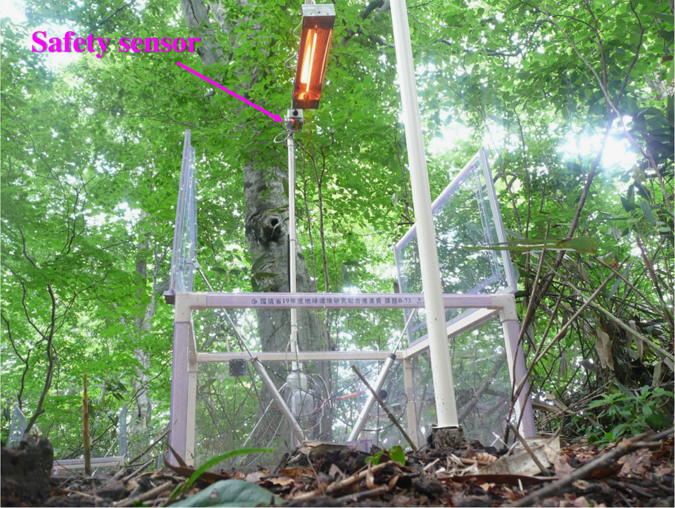
The infrared carbon-filament heat lamp was coupled with a safety sensor during the soil warming experiment in forest to reduce the risk of fire.

Researchers, particularly those in Japan’s Forest Agency, were concerned about the risk of forest fires caused by the infrared heaters. To reduce the risk, we developed a safety sensor that could automatically turn off the lamps when they were shaken by a strong wind or struck by falling tree branches, or when the support poles inclined to about 60° from the horizontal (Fig. 2).

### Multichannel automated chamber system

**Figure 3:**
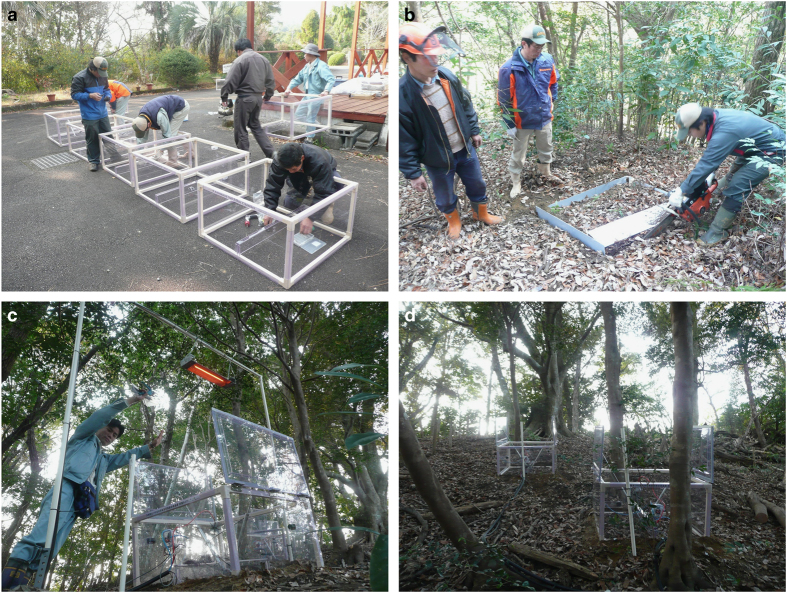
Chamber system installation at the Miyazaki Experimental Warming Site. (**a**) The chambers were assembled in the field. (**b**) Trenches were created using a chainsaw to sever roots. (**c**) The carbon-filament heat lamp was installed above the warming chamber. (**d**) Photograph of the study site with the chambers installed. The authors affirm that the individuals depicted herein provided informed consent for the use of their images.

*R*_s_ is usually measured by means of chamber-based techniques. Liang *et al.*^30–32^ designed a multichannel automated chamber system that applied a flow-through, non-steady-state design to measure *R*_s_ in all four seasons. Briefly, the system comprised a control unit inside a field-accessible case and up to 24 automated chambers. The control unit’s main components were a datalogger (CR1000, Campbell Scientific Inc., Logan, UT, USA), two valve-manifold (CKD-LAC-V-4SB010, CKD Corp., Nagoya, Japan), a micro infrared gas analyser (IRGA; LI-820, LI-COR, Lincoln, NE, USA), and a home-made 62-differential-channel sensor multiplexer. The chambers (90 cm long×90 cm wide×50 cm tall) were constructed of clear PVC plastic sheets (2 mm thick) glued to an aluminium frame (Fig. 3). To minimize the chamber’s edge effects and accurately estimate ecosystem *R*_s_, we designed the chamber size to be as large as possible^31^. To our knowledge, the chamber system used in this study is the largest in the world, and we used the largest number of chambers (15) ever reported for the measurement of *R*_s_. The chamber lids were raised and closed by two pneumatic cylinders (CKD-LAC-C-20B, CKD Corp.) that operated at a pressure of about 0.2 MPa, which was generated by a micro-compressor (0.2LP-7s, Hitachi Ltd., Tokyo, Japan). Between measurements, the two sections of the chamber lid were raised to allow precipitation and leaf litter to reach the enclosed soil surface, thus keeping the soil conditions as natural as possible. During the measurements, the chamber lid was closed and the chamber air was mixed by two micro fans (MF12B, Kyoei Tsushin Kogyo Ltd., Tokyo, Japan). The position and input voltage of the fans were regulated to match the ambient wind speed at the forest floor (usually between 0.1 and 0.2 m s^−1^ at 2 cm above the soil surface)^30,31^. The chamber air was circulated through the IRGA by a 5 l min^−1^ diaphragm pump (CM-50, Enomoto Micropump Ltd., Tokyo, Japan), and the change in the CO_2_ concentration over time was measured by the IRGA. Opening and closing of the chambers were controlled by a home-made relay board that was controlled by the CR1000 datalogger.

At the Miyazaki Experimental Warming Site, we installed 15 chambers. The chambers were distributed randomly on the forest floor within a circular area 40 m in diameter. The polyurethane tubes (4-mm inner diameter; Type U2-6-4, Nitta Moor, Tokyo, Japan) used to withdraw the sampled air from each chamber were all the same length (20 m) to equalize the resistance to flow and ensure the same lag time for all chambers. The chambers were divided into three groups, each with five chambers. The first group was used to measure *R*_s_. The second group was used to measure *R*_h_ by installing the chambers in 1 m×1 m root exclusion (trenched) plots. The third group was used to measure the warming-manipulated *R*_h_ (i.e., *R*_hw_) by installing the chambers in trenched plots with soil warming performed by the carbon-filament heat lamps. Trenches 0.5 to 1 cm wide by 40 cm deep were dug along the plot boundaries with a root-cutting chainsaw (CSVN671AG, Kioritz Co. Ltd., Tokyo, Japan) and then PVC sheets (4 mm thick) were installed in the trenches to a depth of 30 cm to prevent the ingrowth of roots (Fig. 3b). To accurately measure *R*_s_, *R*_h_ and *R*_hw_, the understory vegetation inside the chambers were clipped every 2 weeks when we conducted the field maintenances during the growing season.

## Data Records

### Parameters for efflux calculations

Over the course of an hour, the 15 chambers were closed sequentially and the sampling period for each chamber was 240 s. Therefore, the chambers were open for 93% of the time: during each 1-h cycle each chamber was open for 56 min and closed for 4 min. Thus, most of the rainfall and leaf litter could enter the chambers, and the interior of each chamber had good exposure to any atmospheric turbulence. Air temperature at about 25 cm above the ground and soil temperature at 5 cm below the soil surface were measured inside each chamber with home-made T-type and E-type thermocouples, respectively, that were linked to the sensor multiplexer. To detect any problems related to the sensor multiplexer, we installed air temperature and soil temperature sensors in two selected chambers from the control and warming treatments; these sensors were directly connected to the datalogger. To accurately record the soil temperature, we enclosed the head of the soil temperature sensor in a copper pipe (50 mm long, 6 mm in diameter, and 4 mm in inner diameter) to prevent spatial noise that results from non-homogeneous soil porosity. Moreover, we installed a humidity-temperature sensor (HMP45D, Vaisala, Helsinki, Finland) at a height of 50 cm above the soil surface at the study site to monitor ambient air temperature and relative humidity. This air temperature was used as the air temperature for efflux calculations when the air temperature data from a specific chamber were missing.

Air flow rates through the IRGA were monitored using a high-precision flow transducer (FMS-V001, CKD). In July 2009, to improve the accuracy of the efflux calculations, we began monitoring air pressure at a height of 30 cm in the centre of the measurement plot using a high-precision pressure transducer (PX2760, Omega Engineering, Inc., Stamford, CT, USA). To calculate the components of *R*_s_, the datalogger acquired data from the pressure transducer simultaneously with CO_2_ data from the IRGA at 1-s intervals, and recorded the averaged values every 10 s. Data used for the efflux calculations (hereafter, the ‘efflux datasets’) are available in the comma-delimited text files Miyazaki+yyyy+mm.dat, where yyyy represents the year and mm represents the numerical value of the month [Data Citation 1].

### Additional parameters

Volumetric soil moisture at a depth of 10 cm was monitored for each chamber using time-domain reflectometry (TDR) sensors (EC-5, Decagon Devices, Pullman, WA, USA). However, these sensors proved to be incompatible with the sensor multiplexer. Therefore, in July 2009, we added two TDR sensors (CS616, Campbell Scientific) in a selected trenched chamber and a warming chamber, respectively. To analyse the influence of soil temperature and soil moisture on soil CO_2_ efflux, the datalogger recorded half-hourly mean values from all sensors via the data multiplexer. Data for the efflux-related environmental parameters (hereafter, the ‘environment datasets’) are available in the comma-delimited text files Miyazaki_Environ_yyyy.dat, where yyyy represents the year [Data Citation 1].

## Technical Validation

**Figure 4:**
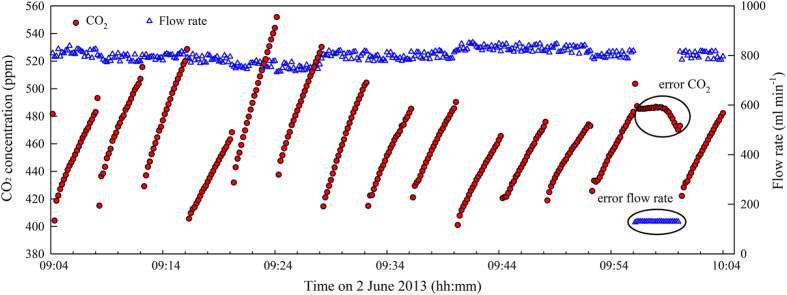
Example of the changes in the CO_2_ concentrations inside the 15 chambers and flow rates through the IRGA during the 1-h measurement cycle. In theory, the CO_2_ concentration inside a chamber should increase linearly. However, the CO_2_ concentration from chamber 14 (red dots enclosed in an ellipse) remained almost constant and then decreased because the airflow through the IRGA stopped (blue open triangles enclosed in an ellipse). This error was caused either by stoppage of the air sampling pump or closure of the valve for chamber 14.

The supporting data and the original data were verified using a number of approaches:

1. **Tree census:** In June 2014, stand density, DBH (>2.0 cm), and canopy height were measured in a 1-ha plot. The forest’s basal area was derived as the sum of the basal areas for each tree, which were calculated as π×(DBH/2)^2^.
2. **Soil carbon and nitrogen measurements:** In June 2014, we sampled the soil using cores obtained to a depth of 30 cm and measured SOC and total soil nitrogen (TN) concentrations. In August 2016, SOC was only measured in the top 5 cm of the soil of the control and warming plots to estimate SOC after the 6-year warming treatment^22^.
3. **Data quality investigation:** M. Takagi downloaded the data from the automated chamber system and sent it to N. Liang and M. Teramoto at 10-day intervals. Liang and Teramoto checked the original data based on a visual inspection of the time courses of the parameter values (Fig. 4).
4. **Sensor calibration:** The LI-820 CO_2_ analyser was calibrated or replaced every 6 to 12 months. The calibration was processed in laboratory following the manual of LI-820 using standard gases (CO_2_ concentration 0 and 700 ppm). Its infrared light source has a rated lifespan of less than 18 000 h (<750 days). Data was missing from 105 days of the 2,202 measurement days from December 2008 to December 2014, corresponding to 4.8% of the whole measurement period. Most of the missing data resulted from premature death of the IRGA infrared light source.
5. **Collation of the data into files:** Each data file was created from the original raw data. The efflux datasets were separated or combined into 1-month durations, and the environment datasets were separated or combined into 1-year durations.

## Usage Notes

### Efflux calculations from the measured data

Based on the chamber method, the soil CO_2_ efflux rate (*R*_s_, μmol m^−2^ s^−1^) is generally calculated using the following equation:

(1)Rs=P0VRS(Tair+273.15)(∂C∂t+C(1000−W)∂W∂t)

where *P*_0_ is the air pressure (Pa), *V* is the effective chamber-head volume (including the sampling tube and extra path space inside the control unit; m^3^), *R* is the ideal gas constant (8.314 Pa m^3^ K^−1^ mol^−1^), *S* is the measured soil surface area (m^2^), *T*_air_ is the air temperature (°C), *∂C/∂t* is the rate of change in the CO_2_ mole fraction (μmol CO_2_ mol^−1^ s^−1^), *C* is the averaged CO_2_ concentration (μmol mol^−1^), *W* is the water vapour mole fraction (mmol H_2_O mol^−1^), and *∂W/∂t* is the rate of change in the water vapour mole fraction (mmol H_2_O mol^−1^ s^−1^). However, the small change in water vapour that occurs inside the chambers is difficult to monitor accurately due to the 20-m-long sampling tube between the chamber and the IRGA. Indeed, most commercially available IRGAs cannot measure water vapour. In practice, the *W* components of the equation can be ignored because it contributes less than 1% of the estimated *R*_s_. Moreover, *V*/*S* in this system is estimated to be 0.5 m. Thus, equation (1) can be simplified as follows:

(2)Rs=60.14PairTair+273.15∂C∂t

where *P*_air_ is the initial air pressure (kPa). *P*_air_ should be the pressure inside the chamber rather than the pressure in the IRGA cell. This is because the pressure inside the IRGA cell is generally several kPa higher or lower than the atmospheric pressure (about 5 kPa higher for our system), depending on aspects of the system’s design such as the capacity of the air sampling pump and the pump’s installation position. To the best of our knowledge, most of the soil CO_2_ efflux measurement systems use either the IRGA cell pressure or the standard atmospheric pressure (101.32 kPa) for their *R*_s_ calculation. Those approaches can lead to overestimates or underestimates of *R*_s_ by several percent. The error can be more significant when measurements are conducted at high-elevation sites. Our chamber system is designed to maintain the minimum possible pressure difference between the chamber and the ambient environment. Therefore, the air pressure (*P*_air_) measured at a height of 30 cm above the soil surface in the centre of our measurement plots was used in our efflux calculation.

Non-linear models, such as the Hutchinson and Mosier model, are suitable for calculating *R*_s_ from chamber measurement with a sufficiently long enclosure time^33^. However, we saw no concave-downward shape for the CO_2_ increase curves during the 240-s measurement period. Therefore, based on the high-resolution data, equation (2) could be modified as follows:

(3)Rs=60.14PairTair+273.151n∑i=1n−1∂C∂t

or

(4)Rs=60.14PairTair+273.15slopeCO2

**Figure 5:**
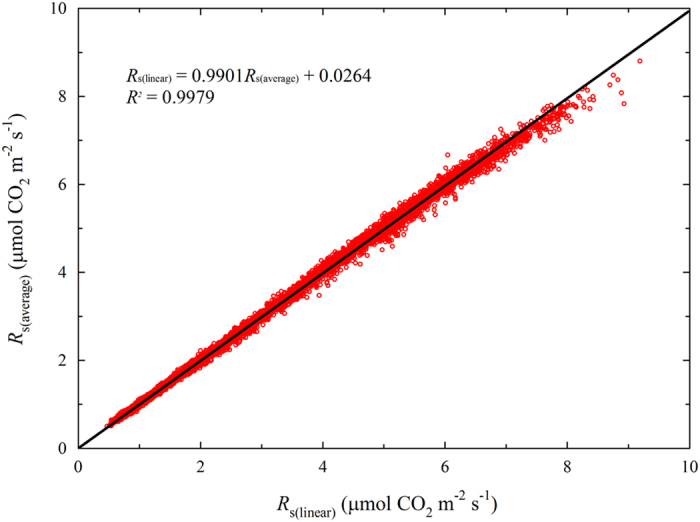
Comparison of the soil respiration (*R*_s_) values estimated using the *average model* (equation (3)) and the *linear model* (equation (4)).

In equation (3), ∂*C*/∂*t* is the rate of change in the CO_2_ mole fraction during each recording interval, and *R*_s_ is estimated from the averaged CO_2_ changes in each data interval (hereafter, the *average model*). In equation (4), *slope*_CO2_ is the slope of the linear fit for all data from each measurement interval for each chamber (hereafter, the *linear model*). Fig. 5 shows hourly *R*_h_ values for the whole year in 2013 for chamber 1 based on both models. The correlation between *R*_h_ values based on the two methods was >0.99, suggesting that both models are suitable for our datasets.

### Suggestions for data processing

**Figure 6:**
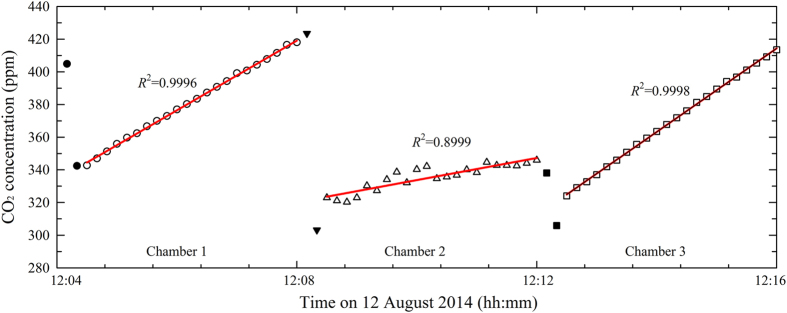
Data quality analysis for identifying outlier data based on triplicate linear regression. The black symbols indicate outliers that were automatically identified through the linear regression.

Ideally, the CO_2_ concentration inside the chambers should increase linearly immediately after the chamber lids are closed. However, several factors could delay this response, including the switching time of the pneumatic cylinders, the flow rate through the sampling tube, and response time of IRGA. We initially regulated the switching time for closing the chamber lids to between 5 and 10 s. It took less than 5 s to pump the chamber air to the IRGA. Thus, the dead band for measuring the CO_2_ concentration from each chamber generally lasted 20 to 30 s for two to three records. The dead band (which includes outlier data) needs to be filtered out. In our studies, we identified outliers by means of linear regression of CO_2_ against the closure time, and used the residuals (the difference between the linear fit and the measured values) to represent the uncertainties in the data, and we repeated the regression twice (Fig. 6)^21,34^. Finally, we used a difference equal to three times the s.d. of the residuals as the criterion for identifying outliers.

**Figure 7:**
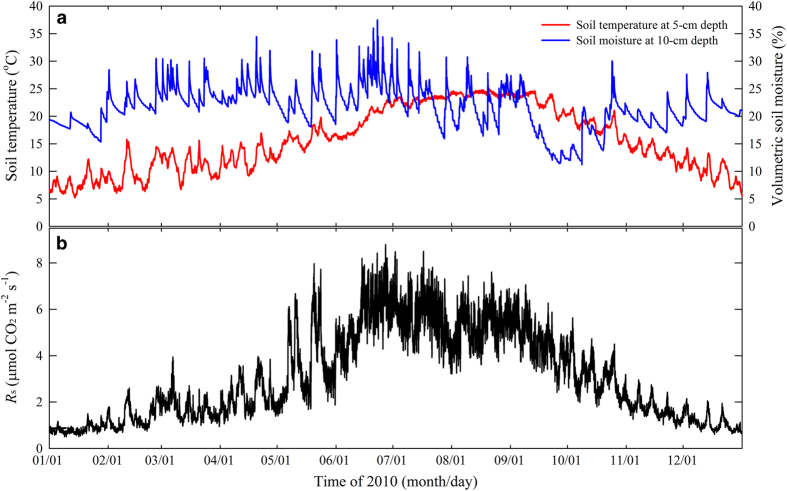
Seasonal changes in (a) soil temperature and soil moisture and (b) soil CO_2_ efflux (*R*_s_). Data in (**a**) were derived from the environment datasets (Data Citation 1); data in (**b**) were derived from the efflux datasets (Data Citation 1).

Several problems could also generate data errors, such as stoppage of the pump that sends the chamber air to the IRGA, times when the chamber lid did not close well or had air leakage or when the micro fan used to create steady mixing of the chamber atmosphere stopped (Fig. 6). When our chamber system is operating normally, the goodness of fit (*R*^2^) for the linear regression of the CO_2_ concentration as a function of time is usually >0.99. However, *R*^2^ is generally <0.90 if any problem occurs with the sampling pump, pneumatic cylinder, or micro fan. Therefore, we usually chose *R*=0.95 as the threshold for detecting usable data. After filtering out the outliers, we used the remaining data to calculate the CO_2_ efflux rates (Fig. 7).

### How many chambers should be used for each treatment?

**Figure 8:**
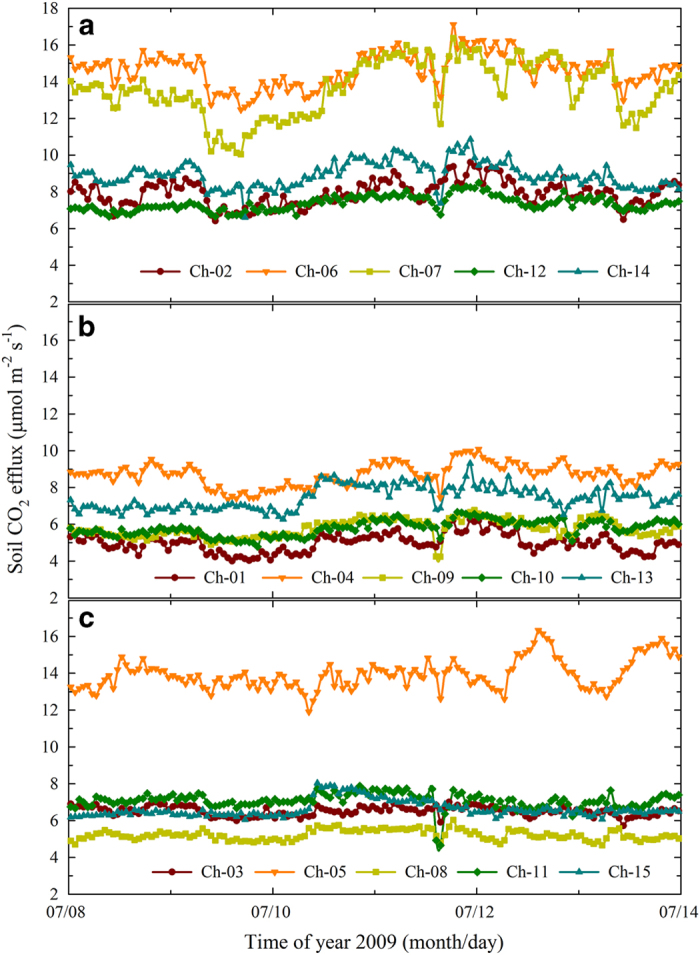
The hourly soil CO_2_ efflux shows high spatial variation based on data from five chambers for each treatment. (**a**) Control treatment (*R*_s_), (**b**) trenched treatment (*R*_h_), and (**c**) warmed trenched treatment (*R*_hw_). Ch, chamber number.

High spatial variation of *R*_s_ values obtained from chamber measurements has been observed in various ecosystems^31,35^. The high variability often exists over a distance of centimetres due to the large heterogeneity in natural soils. The spatial variations are generally controlled by biotic factors such as the spatial structure of fine roots, microbiota, and other soil fauna, as well as by abiotic factors such as the spatial distribution of SOC, soil moisture, topography, and even the sizes of rocks. The spatial variation of *R*_s_ is also associated with the size of the chamber. Though the large chambers we used in this study should have minimized problems associated with small-scale spatial variability of *R*_s_ (ref. 31), we also found initial difference of *R*_s_ within each treatment (Fig. 8). Usually, individual chambers or sampling points are used as the statistical units for estimating a representative mean ecosystem *R*_s_, and the coefficient of variation (CV) is used to represent the spatial variation in *R*_s_. In this study, for example, the mean CV between 8 and 14 July 2009 was 32, 23, and 44% for *R*_s_, *R*_h_, and *R*_hw_, respectively (Fig. 8). In particular, the mean *R*_hw_ was greatly influenced by whether we included chamber 5 (Ch-05) in the analysis (Fig. 8c). However, we believe that the data from chamber 5 was reliable and was not an error caused by mechanical problems, because the efflux value changed dramatically if we moved the collection tube from this chamber to its neighbour, which was located within about 1 m of chamber 5. We also observed a similar phenomenon in other ecosystems using the same measurement protocol, and refer to the points with extremely high efflux points as ‘efflux hot spots’^36^. Therefore, depending on the research objectives, the user of our data can decide how many chambers from each treatment should be included in their analysis.

### Calibration of measured data

Before the start of the warming treatment on 7 January 2009, we conducted an initial measurement between 20 December 2008 and 6 January 2009 (18 days). To analyse the effect of warming on *R*_h_, we first derived the initial coefficients (*Q*_c_) for *R*_h_ and *R*_hw_ using the following equation:

(5)Qc=Rs(all)Rs(trentment)

where *R*_s(all)_ is the mean hourly *R*_s_ (μmol CO_2_ m^−2^ s^−1^) from all the trenched chambers, and *R*_s (treatment)_ represents the mean hourly *R*_s_ for the specific treatment (*R*_h_ and *R*_hw_, respectively) during the same measurement period (18 days). We then used the specific *Q*_c_ to calibrate the initial heterogeneity of the efflux value between *R*_h_ and *R*_hw_. Thus, the warming effect on *R*_h_ (*F*_e_, % °C^−1^) can be estimated as follows:

(6)Fe=QcwRhw−QchRhQchRh(Tsw−Tsh)×100

where *Q*_cw_ and *Q*_ch_ are the *Q*_c_ values for the measured *R*_hw_ and *R*_h_, respectively; and *T*_sw_ and *T*_sh_ are the soil temperatures (°C) in the warming and non-warming treatment plots, respectively. Users of our data are strongly encouraged to adopt the *Q*_c_ calculation to calibrate or modify the measured *R*_h_ and *R*_hw_, particularly for estimation of the effect of warming on *R*_h_.

## Additional Information

**How to cite this article:** Liang, N. *et al.* High-resolution data on the impact of warming on soil CO_2_ efflux from an Asian monsoon forest. *Sci. Data* 4:170026 doi: 10.1038/sdata.2017.26 (2017).

**Publisher’s note:** Springer Nature remains neutral with regard to jurisdictional claims in published maps and institutional affiliations.

## Supplementary Material
